# Editorial: How to circumvent the tumour-promoting effect of cytokine in tumour therapy

**DOI:** 10.3389/fimmu.2023.1298157

**Published:** 2023-10-10

**Authors:** Lingman Ma, Jiakai Hou, Jian Dong, Yanbo Wang, Jifu Wei

**Affiliations:** ^1^ School of Life Science and Technology, China Pharmaceutical University, Nanjing, Jiangsu, China; ^2^ Department of Biology and Biochemistry, University of Houston, Houston, TX, United States; ^3^ Department of Vascular Surgery, The First Affiliated Hospital of Xi'an Jiaotong University, Xi’an, Shaanxi, China; ^4^ School of Life Sciences, Nanjing University, Nanjing, China; ^5^ Jiangsu Cancer Hospital & Jiangsu Institute of Cancer Research & the Affiliated Cancer Hospital of Nanjing Medical University, Nanjing, Jiangsu, China

**Keywords:** tumour-promoting effect, cytokine, tumour therapy, interleukin-32, cytokine signaling in immune-related genes

Cytokines, a class of soluble proteins that are key mediators of cell communication in the tumour microenvironment (TME), can regulate both innate and adaptive immunity (1, 2). Unfortunately, some tumour-related cytokines, especially pro-inflammatory cytokines (such as TNF, IL-6, and IL-1), have a well-recognized tumour-promoting activity and take part in the various phases of tumour initiation and progression (3, 4). Therefore, the research on the tumour-promoting effect of cytokines will play a key role in further revealing the molecular mechanisms of tumour development and identifying novel targets for tumour therapy. In the current Research Topic “*How to Circumvent the Tumour-promoting Effect of Cytokine in Tumour Therapy*”, we attempted to collect the most-recent progress made in tumour-promoting effect of cytokine. In total, after being peer-reviewed, 5 manuscripts, composed with 3 research articles, 1 review article and 1 brief research report authored by 40 researchers worldwide, were successfully accepted for publication.

Interleukin-32 (IL-32) is an important interleukin cytokine usually linked to innate and adaptive immune responses (5). In recent years, it has been found that IL-32 exhibits both pro- and anti- tumour effects. Aass et al. reported that the activation of toll-like receptors (TLRs) in an inflamed or infectious bone marrow microenvironment may promote IL-32 expression in multiple myeloma cells through NF-κB activation, and this may contribute to accelerating the disease. They therefore propose that the subgroup of IL-32-expressing patients may benefit from combination treatments where antibiotics, antiviral- or anti-inflammatory drugs are included. To evaluate the diagnosis and treatment of tumour patients, the prediction value of cytokine signaling in immune-related genes (CSIRGs) is needed. Pu et al. defined a novel CSIRGs signature in melanoma through applying a machine-learning model by single-cell RNA-sequencing datasets. They discovered a 5-CSIRG signature that was substantially related to the overall survival of melanoma patients, which may assist in predicting melanoma patient prognosis, biological characteristics, and appropriate therapy. Autophagy participated in innate immunity, inflammatory response and adaptive immune response by processing antigens and regulating the development and function of lymphocytes (6–8). Based on R software, Yue et al. found that autophagy score and IFNG expression were novel immunotherapy predictive biomarkers, which might play predictive effects through the JAK-STAT signaling pathway. IFNG might be a potential targeted therapy for cisplatin resistant colon cancer and was also a prognostic indicator. Notably, accumulative evidence suggests that the precancerous stem cells (pCSCs)/CSC niche is an inflammatory dominated milieu where contains different cytokines that function as the key communicators between pCSCs/CSCs and their niche and have a decisive role in promoting cancer development, progression, and metastasis (9, 10). Cui et al. presented a systematic review of current and new insights on cytokines, such as interleukin (IL)-4, IL-6, IL-8, IL-17A, IL-22, IL-23, IL-33 and interferon (IFN)-γ, involving in the modulation of pCSC/CSC properties and features in precancerous and cancerous lesions, and discussed the possible mechanisms of adenoma progression to colorectal cancers (CRCs) and their therapeutic potential. Anaplastic large cell lymphoma (ALCL) is characterized by the presence of large anaplastic cells with diffuse and strong expression of CD30 on the cell membrane and in the Golgi region (11). Xiang et al. reported that pSTAT3-Y705/S727 can be used to help distinguish ALK-negative ALCL from CD30^high^ PTCL, NOS, and pSTAT3-S727 expression by tumour infiltrating lymphocytes could predict the prognosis of a subset of PTCL, NOS.

In conclusion, increasing evidence shows that cytokines are heavily involved in regulating both pro- and anti- tumour activities, such as immune activation and suppression, inflammation, cell damage, angiogenesis, cancer stem-cell-like cell maintenance, invasion, metastasis and etc. New insights into the prognosis and treatment of tumour patients may be provided by the in-depth study of tumour-related cytokines.

## Author contributions

LM: Conceptualization, Writing – original draft, Writing – review & editing. JH: Conceptualization, Writing – original draft. JD: Conceptualization, Writing – original draft. YW: Conceptualization, Writing – original draft. JW: Conceptualization, Writing – original draft.

## References

[1] Holicek P Guilbaud E Klapp V Truxova I Spisek R Galluzzi L. Type I interferon and cancer. Immunol Rev (2023). doi: 10.1111/imr.13272 37667466

[2] Salkeni MA Naing A. Interleukin-10 in cancer immunotherapy: from bench to bedside. Trends Cancer (2023) 9:716–25. doi: 10.1016/j.trecan.2023.05.003 PMC10524969 37321942

[3] Cornwell AC Tisdale AA Venkat S Maraszek KE Alahmari AA George A. Lorazepam stimulates IL6 production and is associated with poor survival outcomes in pancreatic cancer. Clin Cancer Res (2023) 29:3793–812. doi: 10.1158/1078-0432.CCR-23-0547 PMC10502465 37587561

[4] Qi D Lu M Xu P Yao X Chen Y Gan L. Transcription factor ETV4 promotes the development of hepatocellular carcinoma by driving hepatic TNF-alpha signaling. Cancer Commun (Lond) (2023). doi: 10.1002/cac2.12482 PMC10693303 37670477

[5] Hough JT Zhao L Lequio M Heslin AJ Xiao H Lewis CC. IL-32 and its paradoxical role in neoplasia. Crit Rev Oncol Hematol (2023) 186:104011. doi: 10.1016/j.critrevonc.2023.104011 37105370

[6] Bai Z Peng Y Ye X Liu Z Li Y Ma L. Autophagy and cancer treatment: four functional forms of autophagy and their therapeutic applications. J Zhejiang Univ Sci B (2022) 23:89–101. doi: 10.1631/jzus.B2100804 35187884 PMC8861559

[7] Bai Z Zhou Y Peng Y Ye X Ma L. Perspectives and mechanisms for targeting mitotic catastrophe in cancer treatment. Biochim Biophys Acta Rev Cancer (2023) 1878:188965. doi: 10.1016/j.bbcan.2023.188965 37625527

[8] Zuo H Chen C Sa Y. Therapeutic potential of autophagy in immunity and inflammation: current and future perspectives. Pharmacol Rep (2023) 75:499–510. doi: 10.1007/s43440-023-00486-0 37119445 PMC10148586

[9] Shi RR Liu J Zou Z Qi YM Zhai MX Zhai WJ. The immunogenicity of a novel cytotoxic T lymphocyte epitope from tumor antigen PL2L60 could be enhanced by 4-chlorophenylalanine substitution at position 1. Cancer Immunol Immunother (2013) 62:1723–32. doi: 10.1007/s00262-013-1478-7 PMC11029738 24077852

[10] Liu P Tang Q Chen M Chen W Lu Y Liu Z. Hepatocellular senescence: immunosurveillance and future senescence-induced therapy in hepatocellular carcinoma. Front Oncol (2020) 10:589908. doi: 10.3389/fonc.2020.589908 33330071 PMC7732623

[11] Thompson MA Stumph J Henrickson SE Rosenwald A Wang Q Olson S. Differential gene expression in anaplastic lymphoma kinase-positive and anaplastic lymphoma kinase-negative anaplastic large cell lymphomas. Hum Pathol (2005) 36:494–504. doi: 10.1016/j.humpath.2005.03.004 15948116

